# Myeloid/lymphoid neoplasms with eosinophilia and tyrosine kinase gene fusions: reevaluation of the defining characteristics in a registry-based cohort

**DOI:** 10.1038/s41375-023-01958-1

**Published:** 2023-07-15

**Authors:** Georgia Metzgeroth, Laurenz Steiner, Nicole Naumann, Johannes Lübke, Sebastian Kreil, Alice Fabarius, Claudia Haferlach, Torsten Haferlach, Wolf-Karsten Hofmann, Nicholas C. P. Cross, Juliana Schwaab, Andreas Reiter

**Affiliations:** 1 Department of Hematology and Oncology, University Hospital Mannheim, Heidelberg University, Mannheim, Germany; 2 Munich Leukemia Laboratory, Munich, Germany; 3 Wessex Regional Genetics Laboratory, Salisbury, UK; 4 Faculty of Medicine, University of Southampton, Southampton, UK

**Keywords:** Disease genetics, Cancer genetics

## Abstract

In a registry-based analysis of 135 patients with “myeloid/lymphoid neoplasms with eosinophilia and tyrosine kinase gene fusions” (MLN-TK; *FIP1L1::PDGFRA*, *n* = 78; *PDGFRB*, diverse fusions, *n* = 26; *FGFR1*, diverse, *n* = 9; *JAK2*, diverse, *n* = 11; *ETV6::ABL1*, *n* = 11), we sought to evaluate the disease-defining characteristics. In 81/135 (60%) evaluable patients, hypereosinophilia (>1.5 × 10^9^/l) was observed in 40/44 (91%) *FIP1L1::PDGFRA* and 7/7 (100%) *ETV6::ABL1* positive patients but only in 13/30 (43%) patients with *PDGFRB*, *FGFR1,* and *JAK2* fusion genes while 9/30 (30%) patients had no eosinophilia. Monocytosis >1 × 10^9^/l was identified in 27/81 (33%) patients, most frequently in association with hypereosinophilia (23/27, 85%). Overall, a blast phase (BP) was diagnosed in 38/135 (28%) patients (myeloid, 61%; lymphoid, 39%), which was at extramedullary sites in 18 (47%) patients. The comparison between patients with *PDGFRA*/*PDGFRB* vs. *FGFR1*, *JAK2,* and *ETV6::ABL1* fusion genes revealed a similar occurrence of primary BP (17/104, 16% vs. 8/31 26%, *p* = 0.32), a lower frequency (5/87, 6% vs. 8/23, 35%, *p* = 0.003) of and a later progression (median 87 vs. 19 months, *p* = 0.053) into secondary BP, and a better overall survival from diagnosis of BP (17.1 vs. 1.7 years, *p* < 0.0008). We conclude that hypereosinophilia with or without monocytosis and various phenotypes of BP occur at variable frequencies in MLN-TK.

## Introduction

The recently published World Health Organization (WHO) 2022 classification and the International Consensus Classification of Myeloid and Lymphoid Neoplasms (ICC-MLN) define a distinct subcategory of myeloid neoplasms as “myeloid/lymphoid neoplasms with eosinophilia and tyrosine kinase gene fusions” (MLN-TK) [1, 2]. This category name has changed from the previous “myeloid/lymphoid neoplasms with eosinophilia and rearrangement of *PDGFRA*, *PDGFRB* or *FGFR1*, or with *PCM1::JAK2*” (MLN-eo) [3], to specify the underlying molecular genetic changes and to include cases with *ETV6::ABL1*, *FLT3* fusions or other TK fusion genes. MLN-TK are driven by rearrangements/fusion genes with involvement of *PDGFRA*, *PDGFRB*, *FGFR1*, *JAK2*, *ABL1,* or *FLT3*. This definition implicates eosinophilia as a recurrent finding, which therefore serves as the main trigger for initiation of distinct cytogenetic and molecular analyses conferring to the identification of disease-defining underlying TK fusion genes. Beside eosinophilia, blast phase (BP) in bone marrow (BM) or at extramedullary sites (extramedullary disease, EMD) of myeloid or lymphoid origin, initially often diagnosed as “myelosarcoma” or “high-grade lymphoma”, is present at diagnosis (primary BP) or develops during follow-up (secondary BP).

To date, more than 70 different TK fusion genes with recurrent involvement of at least six TK (*PDGFRA*, *PDGFRB*, *FGFR1*, *JAK2*, *ABL1*, *FLT3*) have been identified in clinically and morphologically distinct MLN with or without eosinophilia [4]. Targeted treatment with TK inhibitors (TKI) such as imatinib is highly effective in patients with *PDGFRA* and *PDGFRB* fusion genes, e.g., *FIP1L1::PDGFRA* or *ETV6::PDGFRB* [5–8], resulting in excellent long-term survival. In contrast, TK fusion genes with involvement of *FGFR1* or *JAK2* are associated with a more aggressive phenotype and clinical course with variable sensitivity to currently available TKI [4, 9–14].

Single case reports and small series described absence of eosinophilia and/or presence of monocytosis in association with distinct TK fusion genes. Due to the absence of eosinophilia, the diagnosis of a TK-fusion driven MLN may therefore be delayed or even completely missed. Within the “German Registry for Disorders of Eosinophils and Mast Cells (GREM)”, we sought to evaluate incidence, the incidence of eosinophilia and monocytosis and the phenotype and prognosis of BP within this distinct subcategory of myeloid neoplasms.

## Patients and methods

### Patients

Within the GREM, we identified 135 patients with diagnosis of a MLN-TK. The involved TK included *PDGFRA* (all *FIP1L1::PDGFRA* positive, *n* = 78), *PDGFRB* (diverse fusion partners, *n* = 26), *FGFR1* (diverse fusion partners, *n* = 9), *JAK2* (*PCM1* or *BCR* as fusion partners, *n* = 11) and *ABL1* (*ETV6::ABL1*, *n* = 11, Table 1). Patients with primary *ETV6::ABL1* positive ALL were not included. The 135 patients were recruited from approximately 60 participating hematology centers and hematologists in private practice. Fifteen patients had been diagnosed with a suspected eosinophilia-associated myeloid neoplasm prior to 2002 and were subsequently tested *FIP1L1::PDGFRA* positive in 2003. Sixty-seven patients were recruited between 2003 and 2012, 53 patients between 2013 and 2022. We repeatedly reported on treatment of various MLN-TK with specific TKI, e.g., *PDGFRA*/*PDGFRB* fusion genes with imatinib [7, 15], *JAK2* fusion genes with ruxolitinib [10, 16] and *ETV6::ABL1* fusion gene with imatinib, nilotinib and dasatinib [16].

**Table 1:** Patients’ characteristics of 135 patients with myeloid/lymphoid neoplasms with eosinophilia and tyrosine kinase fusion genes.

	No. of patients	Partner genes	Eosinophilia (*n*) >0.5/>1.5 × 10^9^/l (%)	Monocytosis >1 × 10^9^/l	Overall survival
*FIP1L1::PDGFRA*	78	1	44 100%/91%	44 27%	92% at 5 years 92% at 10 years
*PDGFRB*	26	11	16 75%/50%	16 31%	78% at 5 years 78% at 10 years
*FGFR1*	9	3	6 50%/16%	6 33%	57% at 5 years
*JAK2*	11	2	8 75%/50%	8 25%	55% at 5 years
*ETV6::ABL1*	11	1	7 100%/100%	7 85%	58% at 4 years

In the current analysis, OS was analyzed in the cohort of 135 patients (male 126/135; median age 49 years, range 19–80), in either chronic phase (CP) from time of diagnosis (*n* = 110, including 13 patients with progression to secondary BP) or in BP from time of diagnosis of BP (*n* = 38, primary BP, *n* = 25; secondary BP, *n* = 13). Of note, the 13 patients with secondary BP are included in both cohorts (Table 2). Data on absolute and relative counts of eosinophils and monocytes at time of diagnosis were available for 81 patients: *FIP1L1::PDGFRA* (*n* = 44), *PDGFRB* (*n* = 16), *FGFR1* (*n* = 6), *JAK2* (*PCM1::JAK2*, *n* = 7, *BCR::JAK2*, *n* = 1) or *ETV6::ABL1* (*n* = 7, Table 1). All patients gave written informed consent. Data collection was compliant with the Declaration of Helsinki and approved by the ethics committee of the Medical Faculty Mannheim at the University Heidelberg, Germany.

**Table 2:** Phenotype of blast phase in 38 patients diagnosed with myeloid/lymphoid neoplasms with eosinophilia and tyrosine kinase fusion genes (*n* = 135).

Fusion gene	*n*	Primary BP	CP at diagnosis	Secondary BP	Myeloid		Lymphoid	
					BM (primary/secondary)	EMD (primary/secondary)	BM (primary/secondary)	EMD (primary/secondary)
*FIP1L1::PDGFRA*	78	13	65	4	10 (8/2)	4 (3/1)	–	3 (2/1)
*PDGFRB*^a^	26	4	22	1	1 (0/1)	1 (1/0)	1 (1/0)	2 (2/0)
*FGFR1*^a^	9	6	3	1	–	2 (2/0)	2 (1/1)	3 (3/0)
*JAK2*^a^	11	0	11	3	1 (0/1)	–	2 (0/2)	–
*ETV6::ABL1*	11	2	9	4	2 (0/2)	2 (1/1)	1 (0/1)	1 (1/0)
Overall	135	25	110	13	14 (8/6)	9 (7/2)	6 (2/4)	9 (8/1)

*BM* bone marrow, *CP* chronic phase, *BP* blast phase, *EMD* extramedullary disease.

^a^Various fusion partners.

### Cytogenetics and molecular analyses

Cytogenetics and fluorescence in situ hybridization (FISH) analyses were performed on BM according to standard procedures. Specific nested reverse transcription polymerase chain reaction (RT-PCR) was performed for confirmation of suspected fusion genes in all patients [9, 17, 18].

### Statistical analyses

All clinical and laboratory parameters including peripheral blood cell counts are expressed as median and range. Overall survival (OS) was determined from date of diagnosis to date of death or last contact and calculated by using the Kaplan–Meier method. Pearson correlation analysis was performed for the correlation between two parameters. Differences in the distribution of continuous variables between categories were analysed by Mann–Whitney test (for comparison of two groups). For categorical variables, Fisher’s exact test was used. *P* < 0.05 (two-sided) were considered significant. All statistical analyses were performed using GraphPad Prism Software, Inc. version 7 and SPSS (version 28.0; IBM-Corporation, Armonk, NY, USA).

## Results

### Incidence and phenotype of blast phase

A BP of myeloid (23/38, 61%) or lymphoid (15/38, 39%) origin was diagnosed in 38/135 (28%) patients (Tables 1 and 2). BP in BM (≥20% blast cells, *n* = 20, 53%; myeloid, *n* = 14; lymphoid, *n* = 6) was primary in 10/20 (50%) or secondary in 10/20 (50%) patients while EMD (*n* = 18) was primary in 15/18 (83%; myeloid, *n* = 7; lymphoid, *n* = 8) or secondary in only 3/18 patients (17%; myeloid, *n* = 2; lymphoid, *n* = 1, Table 3). Independent of phenotype or time point of occurrence of EMD, the phenotype in BM or peripheral blood (PB) was myeloid in all cases. A lineage discordance between BM/PB and EMD was therefore observed in 9/18 (50%) patients (Table 2). Compared to patients with *FGFR1*, *JAK2* and *ETV6::ABL1* fusion genes, *PDGFRA*/*PDGFRB* fusion positive patients had a lower frequency of primary (17/104, 16% vs. 8/31 26%, *p* = 0.32) and secondary BP (5/87, 6% vs. 8/23, 35%, *p* = 0.003), which occurred at later time points (median 87 months, range 9–189 vs. 19 months, range 10–36; *p* = 0.053).

**Table 3:** Anatomic localization and phenotype of histologically confirmed myeloid and lymphoid extramedullary disease (EMD) in 18 patients.

Anatomic localization	Myelosarcoma (M) Lymphoma	Primary	Secondary	FISH
*FIP1L1::PDGFRA*				
Femur	M	x		x
Pharynx	M	x		
Bone, meningeal	M	x		
Lymph nodes, paraspinal	M		x	x
Lymph nodes	T-LBL	x		x
Lymph nodes	T-LBL	x		x
Lymph nodes	T-LBL		x	x
*PDGFRB*				
Pleural, cerebral	M	x		x
Lymph nodes, liver	T-LBL	x		
Lymph nodes	T-cell lymphoma	x		
*FGFR1*				
Lymph nodes, bladder	M	x		
Lymph nodes, bone, spine	M	x		x
Lymph nodes	T-LBL	x		
Lymph nodes	T-LBL	x		
Lymph nodes	T-LBL	x		
*ETV6::ABL1*				
Parotid gland, bone (multiple sites)	M	x		
Humerus	M		x	
Lymph nodes	T-LBL	x		

In several cases, the presence of the fusion gene was confirmed by FISH analysis.

*M* myeloid, *T-LBL* T-lymphoblastic lymphoma.

### Survival

**Fig. 1:**
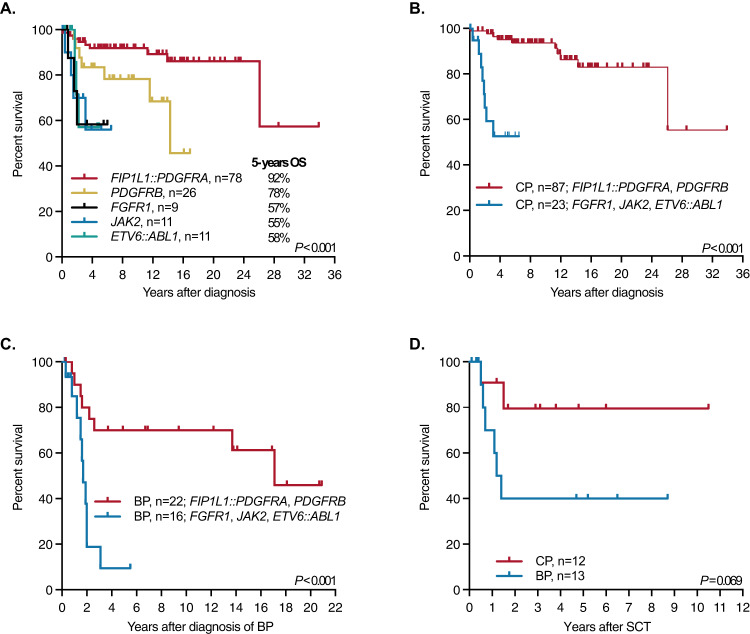
Overall surviall of all patients. **A** Overall survival (OS) from time of diagnosis of 135 patients with various tyrosine kinase fusion genes (*PDGFRA*, *n* = 78*; PDGFRB*, *n* = 26; *FGFR1*, *n* = 9; *JAK2*, *n* = 11; *ETV6::ABL1*, *n* = 11) independent of disease phase. **B** OS of patients with *PDGFRA*/*PDGFRB* (*FIP1L1::PDGFRA*, *n* = 65; *PDGFRB*, *n* = 22) and *FGFR1*, *JAK2* and *ETV6::ABL1* (*FGFR1*, *n* = 3; *JAK2*, *n* = 11; *ETV6::ABL1*, *n* = 9) fusion genes in chronic phase (including 13 patients with progression into secondary blast phase), median follow-up 9.7 years (0–34.0) and 2.2 years (0.1–6.3), respectively (*p* < 0.0001). **C** OS of patients with *PDGFRA*/*PDGFRB* (*FIP1L1::PDGFRA*, *n* = 17; *PDGFRB*, *n* = 5) and *FGFR1*, *JAK2* and *ETV6::ABL1* (*FGFR1*, *n* = 7; *JAK2*, *n* = 3; *ETV6::ABL1*, *n* = 6) fusion genes from diagnosis of blast phase (primary BP, *n* = 25, secondary BP, *n* = 13), median OS 17.1 years (range 0.2–22) vs. 1.7 years (range 0.1–5.5; *p* = 0.0008). **D** OS of patients after allogeneic stem cell transplantation in prior chronic (*n* = 12) or blast phase (*n* = 13) independent of underlying TK fusion gene.

In MLN with *PDGFRA*/*PDGFRB* fusion genes, 16/104 (15%) patients had died after a median follow-up of 9.2 years (range 0–28.6, Fig. 1A, B). While in CP (*n* = 87), 8 patients died because of comorbidity (*n* = 5; *PDGFRA*, *n* = 2, *PDGFRB*, *n* = 3), resistance/progression (*n* = 1; *PDGFRB*, *n* = 1), resistance/allogeneic SCT/GvHD (*n* = 1) and cardiac involvement (*n* = 1). Five patients progressed into secondary BP (*PDGFRA*, *n* = 4, *PDGFRB*, *n* = 1). Causes of death in BP (*n* = 8; primary, *n* = 6; secondary, *n* = 2) included resistance/relapse (*n* = 4; *PDGFRA*, *n* = 2, *PDGFRB*, *n* = 2), comorbidity (*n* = 3; *PDGFRA*, *n* = 2, *PDGFRB*, *n* = 1) and intracerebral bleeding (*n* = 1; *PDGFRA*, *n* = 1). Mutations conferring resistance to imatinib were identified in 2 patients (*PDGFRA* T674I, *n* = 2).

In MLN with *FGFR1*, *JAK2* and *ETV6::ABL1* fusion genes (*n* = 31; BP, *n* = 16), 11 patients died of which 10 patients were previously diagnosed with BP. The median OS from diagnosis of BP was 1.7 years (range 0.1–5.5, Fig. 1C). Overall, the incidence of BP was significantly lower (22/104, 21% vs. 16/31, 52%; *P* = 0.002) and overall survival from diagnosis of BP was significantly better (17.1 vs. 1.7 years, *P* < 0.0008) in patients with *PDGFRA*/*PDGFRB* fusions than with other TK fusion genes (Table 1, Fig. 1C).

### Allogeneic stem cell transplantation

Allogeneic stem cell transplantation (SCT) was performed in 25 patients (Table 4) at a significant lower frequency in patients with *PDGFRA*/*PDGFRB* fusion genes (9/104, 9%, CP, *n* = 3; BP, *n* = 6) than in patients with *FGFR1*, *JAK2* and *ETV6::ABL1* fusion genes (16/31, 52%, CP, *n* = 9; BP, *n* = 7). After allogeneic SCT, 10/12 CP patients are alive at median 3.0 (range 0.3–10.5) years and 2/12 patients (*PDGFRA*, *n* = 1; *JAK2*, *n* = 1) died because of relapse at 0.5 and 1.5 years. In BP, 7/13 patients are alive at median 4.7 (range 0.1–6.5) years and 6/13 patients died at median 0.9 (range 0.6–1.4) years (Fig. 1D).

**Table 4:** Outcome after allogeneic stem cell transplantation according to fusion gene and disease phase (*n* = 25; CP, *n* = 12; BP, *n* = 13).

Fusion gene	Disease phase	Allogeneic SCT		Phenotype	Alive (years after allogeneic SCT)	Death (years after allogeneic SCT)
*FIP1L1:: PDGFRA*	CP	3/65 (4.6%)	9/104 CP: *n* = 3 BP: *n* = 6	- PDGFRA T674I mutation, *n* = 2; unknown (*n* = 1)	- *n* = 2 - +10.5, +1.2	- *n* = 1 (GvHD while in CHR) - +1.5
	BP	3/17 (17.6%)		- Primary myeloid BP in BM (*n* = 3)	- *n* = 2 - +6.5, +4.7	- *n* = 1 (relapse) - +1.4
*PDGFRB*^1^	CP	0/22				
	BP	3/5 (60%)		- Primary lymphoid BP in BM (*n* = 1) - Primary lymphoid EMD (*n* = 1), - Secondary myeloid BP in BM (*n* = 1)	- *n* = 1 - +8.7	- *n* = 2 (relapse) - +1.2, +0.7
*FGFR1*^1^	CP	2/2 (100%)	16/31 CP: *n* = 9 BP: *n* = 7		- *n* = 2 - +2.9, +6.0	
	BP	4/7 (57.1%)		- Primary lymphoid in EMD (*n* = 3) - Primary myeloid in EMD (*n* = 1)	- *n* = 3 - +5.2, +0.4, +0.3	- *n* = 1 (relapse) - +1.1
*JAK2*^1^	CP	6/11 (54.5%)			- *n* = 5 - +0.5, +1.7, +3.1, +3.8, +4.8	- *n* = 1 (relapse) - +0.5
	BP	1/3 (33.3%)		- Secondary lymphoid BP in (*n* = 1)		- *n* = 1 (relapse) - +0.6
*ETV6:: ABL1*^1^	CP	1/9 (11.1%)			- *n* = 1 - +0.3	
	BP	2/6 (33.3%)		- Secondary myeloid BP (*n* = 1) in BM or EMD (*n* = 1)	- *n* = 1 - +0.1	- *n* = 1 (GvHD while in CMR) - +0.6
Overall		25				

*BP* blast phase, *CHR* complete hematologic remission, *CMR* complete molecular remission, *CP* chronic phase, *GvHD* Graft verus Host Disease, *SCT* stem cell transplantation.

### Age, gender, partner genes, and eosinophilia in association with various TK fusion genes

#### *FIP1L1::PDGFRA*

At diagnosis, the median age was 45.5 years (range 19–70), 43/44 (98%) patients were male. Leukocytosis >10 × 10^9^/l was present in 29/44 (66%) patients (median 14 × 10^9^/l, range 5–156), eosinophilia (median 6.4 × 10^9^/l, range 0.9–30.1) was >0.5 × 10^9^/l or >1.5 × 10^9^/l in 44/44 (100%) and 40/44 (90%) patients, respectively (Table 5).

**Table 5:** Frequency of eosinophilia and monocytosis in myeloid/lymphoid neoplasms with eosinophilia and tyrosine kinase fusion gens (MLN-TK, *n* = 81) in relation to various fusion genes.

Fusion gene	*n*	Eosinophils				Monocytes >1 × 10^9^/l		
		×10^9^/l median, (range)	≤0.5 × 10^9^/l	>0.5–1.5 × 10^9^/l	>1.5 × 10^9^/l	*n*	Eosinophils 0.5–1.5 × 10^9^/l	Eosinophils >1.5 × 10^9^/l
*FIP1L1::PDGFRA*	44	6.4 (0.9–30.1)	0/44	4/44 (9%)	40/44 (91%)	12/44 (27%)	–	12/12
*PDGFRB*^a^	16	1.6 (0.2–12.0)	4/16 (25%)	4/16 (25%)	8/16 (50%)	5/16 (31%)	1/5	3/5
*FGFR1*^a^	6	0.6 (0–2.5)	3/6 (50%)	2/6 (33%)	1/6 (16%)	2/6 (33%)	2/2	–
*JAK2*^a^	8	1.5 (0–4.6)	2/8 (25%)	2/8 (25%)	4/8 (50%)	2/8 (25%)	–	2/2
*ETV6::ABL1*	7	5.6 (2.0–7.1)	–	–	7/7 (100%)	6/7 (85%)	–	6/6
Overall	81	–	9/81 (11%)	12/81 (15%)	60/81 (74%)	27/81 (33%)	3/27 (11%)	23/27 (85%)

^a^Various fusion partners.

#### *PDGFRB* fusion genes

In 16 patients (male 14/16; median age 53 years, range 20–80), eleven different partner genes of *PDGFRB* were identified. Only *ETV6* was a recurrent fusion partner (*ETV6*, *n* = 5; *CDCC88C*, *n* = 1; *CCDC6*, *n* = 1; *CEP120*, *n* = 1; *CPSF6*, *n* = 1; *GIT2*, *n* = 1; *GPIAP1*, *n* = 1; *MYO18A*, *n* = 1; *PRKG2*, *n* = 1; *SPECC1*, *n* = 1; *TP53BP1*, *n* = 1; uncharacterized partner, *n* = 1). Leukocytosis >10 × 10^9^/l was present in 13/16 (81%) patients (median 28 × 10^9^/l, range 4–127). Eosinophils (median 1.6 × 10^9^/l, range 0.2–12.0) were ≤0.5 × 10^9^/l, >0.5 × 10^9^/l and >1.5 × 10^9^/l in 4/16 (25%), 12/16 (75%) and 8/16 (50%) patients, respectively.

#### *FGFR1* fusion genes

In six patients with *FGFR1* fusion genes (male 5/6; median age 58 years, range 49–77), *ZMYM2* (*n* = 3) and *BCR* (*n* = 2) were recurrent partner genes (*FGFR1OP::FGFR1*, *n* = 1). Leukocytosis >10 × 10^9^/l was observed in 5/6 (83%) patients (median 64.5 × 10^9^/l, range 4.8–173.0). Eosinophils (median 0.6 × 10^9^/l, range 0–2.5) were ≤0.5 × 10^9^/l, >0.5 × 10^9^/l and >1.5 × 10^9^/l in 3/6 (50%), 3/6 (50%) and 2/6 (33%) patients, respectively.

#### *JAK2* fusion genes

All 8 patients with *JAK2* fusion genes (*PCM1*, *n* = 7; *BCR*, *n* = 1; male 7/8; median age 69 years, range 29–73) presented with leukocytosis (median 25.9 × 10^9^/l, range 10.5–55.0). Eosinophils (median 1.5 × 10^9^/l, range 0–4.6) were ≤0.5 × 10^9^/l, >0.5 × 10^9^/l and >1.5 × 10^9^/l in 2/8 (25%), 6/8 (75%) and 4/8 (50%) patients, respectively.

#### *ETV6::ABL1* fusion gene

All *ETV6::ABL1* positive patients (*n* = 7; male 6/7; median age 30 years, range 20–74) presented with leukocytosis >10 × 10^9^/l (median 62 × 10^9^/l, range 20.9–143). Hypereosinophilia >1.5 × 10^9^/l (median 5.6 × 10^9^/l, range 2.0–7.1) was observed in 7/7 (100%) cases.

### Hypereosinophilia >1.5 × 10^9^/l

Overall, hypereosinophilia >1.5 × 10^9^/l was observed in 60/81 (74%) evaluable patients, most frequently in patients with *FIP1L1::PDGFRA* (40/44, 90%) and *ETV6::ABL1* (7/7, 100%) fusion genes. In contrast, it was only observed in 13/30 (43%) patients with *PDGFRB*, *FGFR1* or *JAK2* fusion genes Absence of eosinophilia was restricted to 9/30 (30%) patients with *PDGFRB*, *FGFR1* and *JAK2* fusion genes (Table 5). In those 9 patients, primary diagnoses included atypical chronic myeloid leukemia (*PDGFRB*, *n* = 1; *FGFR1*, *n* = 1), myelodysplastic/myeloproliferative neoplasm (*PDGFRB*, *n* = 1; *FGFR1*, *n* = 1), myeloproliferative neoplasm unclassified (*PDGFRB*, *n* = 2; *JAK2* *n* = 1), myelofibrosis (*JAK2*, *n* = 1) and mixed phenotype acute leukemia (*FGFR1*, *n* = 1). In all 9 patients, the underlying fusion gene was indicated by a characteristic reciprocal translocation.

### Monocytosis >1.0 × 10^9^/l

Irrespective of the underlying TK fusion gene, monocytosis >1.0 × 10^9^/l was observed in 27/81 (33%) patients (*FIP1L1::PDGFRA*, 12/44, 27%; *PDGFRB*, 5/16, 31%; *FGFR1*, 2/6, 33%; *JAK2*, 2/8, 25%; *ETV6::ABL1*, *n* = 6/7, 85%) with relative monocytosis ≥10% being present in 6/27 (22%) patients (Table 5). In *FIP1L1::PDGFRA* positive patients, a significant association was noted between the absolute number of eosinophils and monocytes (*r* = 0.52, *p* = 0.0002). Monocytosis was present in 6/7 (85%) *ETV6::ABL1* positive patients, all 6 patients also had hypereosinophilia >1.5 × 10^9^/l. Overall, monocytosis >1.0 × 10^9^/l was significantly associated with hypereosinophilia >1.5 × 10^9^/l (23/27, 85%) but was without significant impact on progression or OS after a median follow-up of 7.2 years (range 0.1–33.1).

### Serum tryptase

Due to the known association between increased basic serum tryptase levels (normal <11.4 µg/l) and *PDGFRA*/*PDGFRB* fusion genes, serum tryptase levels were available from 43/104 *PDGFRA/PDGFRB* positive patients. The serum tryptase level was ≥11.4 µg/l in 31/43 (72%) and ≥20 µg/l in 23/43 (53%) patients, the median level was 22.9 µg/l (range 3–183). The formal need to adjust normal ranges in patients with hereditary alpha-tryptasemia (HaT) could not be performed because none of the patients was retrospectively tested [19].

## Discussion

Common features of the vast majority of TK-fusion driven myeloid neoplasms include an underlying chronic myeloid neoplasm with a high incidence of concurrent primary BP or progression to secondary BP. BP can be myeloid or lymphoid and is identified in the BM or at extramedullary sites (EMD). In the EMD, initial diagnosis frequently states “myelosarcoma” or “T-cell lymphoma”, while in the BM, the differentiation between a de novo myeloid/lymphoid/biphenotypic acute leukemia and a myeloid or lymphoid BP also remains challenging. We have reported on several patients with suspected primary lymphoma or de novo acute leukemia in which the underlying TK fusion gene was only identified because of poor response to intensive chemotherapy or even allogeneic SCT and persisting eosinophilia [7].

In the currently reported cohort of 135 MLN-TK patients, incidence, phenotype and prognosis of BP was highly variable within the various cohorts of MLN-TK. BP occurred equally distributed either in the BM or as EMD in approximately 30% of patients. It was primary in approximately 70% of patients with a lower relative frequency of 16% in patients with *PDGFRA*/*PDGFRB* fusion genes as compared to 26% in patients with *FGFR1*, *JAK2* and *ETV6::ABL1* fusion genes. In patients with *PDGFRA*/*PDGFRB* fusion genes, secondary BP only occurred in 6% of patients after a median of 87 months because >90% of patients achieved durable complete hematologic, complete cytogenetic (*PDGFRB*) and complete molecular (*FIP1L1*::*PDGFRA*) remissions on imatinib.

In contrast, neither ponatinib on *FGFR1* [20], ruxolitinib on *JAK2* [10, 16] nor imatinib/nilotinib/dasatinib on *ETV6:ABL1* fusions [16] have shown a similar efficacy than imatinib on *PDGFRA/PDGFRB* fusions (Fig. 1A–C). Of interest, the FIGHT-203 study presented promising results on pemigatinib in patients with *FGFR1* fusions in CP and to a lesser extent in BP [21]. In a recent literature review of a heterogenous cohort of 66 *PCM1::JAK2* positive patients, Kaplan et al. reported on 11 ruxolitinib-treated patients [12]. However, the authors did not draw conclusions on its effect on survival because of the small cohort and because analysis on survival was complicated by the fact that 5 of these patients received a subsequent allogeneic SCT with a 5-year survival of 75% [12]. In consequence, patients with *FGFR1*, *JAK2,* and *ETV6::ABL1* fusion genes progressed more often (35%) and faster (median 19 months) into secondary BP than patients with *PDGFRA*/*PDGFRB* fusion genes. The inferior prognosis of patients with *FGFR1*, *JAK2,* and *ETV6*::*ABL1* fusion genes with a median 5-year survival of approximately 50–60% is therefore related to the more aggressive phenotype and the lack of effective and durable conventional treatment [16, 22–26]. In line with recently published data on patients with *FGFR1* [25] and *JAK2* fusion genes [12], data confirm that the poor prognosis of primary and secondary BP can only be overcome by allogeneic SCT (Table 4 and Fig. 1A–D).

Consistent hypereosinophilia >1.5 × 10^9^/l in more than 90% of patients was only observed in association with *FIP1L1::PDGFRA* and *ETV6::ABL1* fusion genes, although we acknowledge an obvious ascertainment bias in that only cases with eosinophilia are routinely screened for distinct TK fusion genes, particularly *FIP1L1::PDGFRA*. In patients with *PDGFRB* fusion genes, hypereosinophilia >1.5 × 10^9^/l was present in only 50% of patients and even absent (≤0.5 × 10^9^/l) in 25% of patients. Lack of eosinophilia was also evident in patients with *FGFR1* fusions (Table 5). These findings are in line with literature reports on the impact of the *FGFR1* partner gene on phenotype [27]. While *ZMYM2*::*FGFR1* positive patients frequently present with the combination of a T-cell lymphoma/T-ALL/mixed phenotype acute leukemia and eosinophilia, *BCR*::*FGFR1* positive patients usually present with a MPN/CML-like phenotype but a much lower incidence of hypereosinophilia >1.5 × 10^9^/l, in the literature overall only reported in 3/21 evaluable patients [11, 28–50].

Diagnosis of a TK fusion gene driven MLN may be missed due to the lack of eosinophilia and subsequent diverse morphological diagnoses [5]. A significant proportion of patients were not initially diagnosed as MLN-TK or chronic eosinophilic leukemia but rather as subtype of MDS/MPN or MPN unclassified and decisive diagnostic assays such as cytogenetic analysis, FISH analysis or specific RT-PCR were not performed or only with delay. Moreover, newly available NGS technologies such as targeted RNA-sequencing, whole transcriptome or whole genome sequencing have revealed an increasing number of cytogenetically cryptic [51] or cytogenetically difficult to identify fusion genes, e.g., *ETV6::ABL1* and several fusion genes with involvement of *PDGFRB* [52].

A rarely recognized feature of MLN-TK is monocytosis >1.0 × 10^9^/l which was identified in about one third of patients. It was clearly clustered in patients with hypereosinophilia >1.5 × 10^9^/l and consequently in patients with *FIP1L1::PDGFRA* or *ETV6::ABL1* fusion genes. However, only approximately 20% of these patients also had relative monocytosis ≥10%. Even with taking into account the new cut-off values for diagnosis of chronic myelomonocytic leukemia with absolute monocytosis of ≥0.5 × 10^9^/l and relative monocytosis of ≥10%, these numbers did not substantially change. The data, therefore, clearly indicate that a MLN-TK may cause monocytosis but accompanying features include hypereosinophilia and relative monocytosis <10% in the vast majority of patients (Table 5).

Besides MLN-TK, the concurrent presence of significant eosinophilia and monocytosis is also a typical feature in patients with advanced systemic mastocytosis [53, 54]. While being the disease-defining characteristic for chronic myelomonocytic leukemia, monocytosis is also identified in other myeloid neoplasms, potentially as marker of poor prognosis, e.g., in polycythemia vera, myelofibrosis and systemic mastocytosis [55, 56]. These data therefore also underscore the current guidelines for diagnosis of chronic myelomonocytic leukemia, other myelodysplastic/myeloproliferative neoplasms and myeloproliferative neoplasms unclassified that the primary genetic work-up should not only exclude *BCR::ABL1* positive chronic myeloid leukemia but also cases of MLN-TK. Due to the excellent prognosis of imatinib-treated patients with *PDGFRA/PDGFRB* [5, 57, 58] fusion genes, monocytosis had no obvious impact on progression and survival.

*FIP1L1::PDGFRA* and *ETV6::ABL1* fusion genes share striking clinical and morphological similarities including male predominance, the relative frequency of eosinophilia and monocytosis, the median absolute number of eosinophils, and presentation or progression to BP including EMD. Of interest, progression to lymphoid BP in the BM seems to be a rare event for both fusion genes. Compared to *FIP1L1::PDGFRA*, the responses of *ETV6::ABL1* positive patients to imatinib, nilotinib or dasatinib are less frequent and less durable [16]. In the current update of our own cohort, 6/11 patients were initially treated with imatinib but more durable remissions were only observed on primary or secondary treatment with nilotinib (*n* = 2), dasatinib (*n* = 3) or after allogeneic SCT (*n* = 1). Not included in our series, but important to note is that the MLN-TK subcategory also includes very rare fusion genes with involvement of other TK such as *FLT3* [52, 59].

In summary, the relative frequency of the defining characteristics of MLN-TK such as myeloid or lymphoid BP and/or eosinophilia occur at markedly variable frequencies according to the underlying fusion gene. Monocytosis is a potentially important marker which frequently occurs in association with significant eosinophilia. Careful attention must be paid to these subtle characteristics to avoid missing a diagnosis of a TKI-sensitive MLN-TK.

## Data Availability

Freely available to any researcher
